# Estimation of the *Relative* Sensitivity of the Comparative Tuberculin Skin Test in Tuberculous Cattle Herds Subjected to Depopulation

**DOI:** 10.1371/journal.pone.0043217

**Published:** 2012-08-21

**Authors:** Katerina Karolemeas, Ricardo de la Rua-Domenech, Roderick Cooper, Anthony V. Goodchild, Richard S. Clifton-Hadley, Andrew J. K. Conlan, Andrew P. Mitchell, R. Glyn Hewinson, Christl A. Donnelly, James L. N. Wood, Trevelyan J. McKinley

**Affiliations:** 1 Disease Dynamics Unit, Department of Veterinary Medicine, University of Cambridge, Cambridge, United Kingdom; 2 Animal Health and Veterinary Laboratories Agency and Bovine Tuberculosis Programme of the Department for Environment, Food and Rural Affairs, London, United Kingdom; 3 Animal Health and Veterinary Laboratories Agency, West Midlands Regional Office, Stafford, United Kingdom; 4 Animal Health and Veterinary Laboratories Agency, Weybridge, United Kingdom; 5 MRC Centre for Outbreak Analysis and Modelling, Department of Infectious Disease Epidemiology, Imperial College London, London, United Kingdom; University of Ottawa, Canada

## Abstract

Bovine tuberculosis (bTB) is one of the most serious economic animal health problems affecting the cattle industry in Great Britain (GB), with incidence in cattle herds increasing since the mid-1980s. The single intradermal comparative cervical tuberculin (SICCT) test is the primary screening test in the bTB surveillance and control programme in GB and Ireland. The sensitivity (ability to detect infected cattle) of this test is central to the efficacy of the current testing regime, but most previous studies that have estimated test sensitivity (relative to the number of slaughtered cattle with visible lesions [VL] and/or positive culture results) lacked post-mortem data for SICCT test-negative cattle. The slaughter of *entire* herds (“whole herd slaughters” or “depopulations”) that are infected by bTB are occasionally conducted in GB as a last-resort control measure to resolve intractable bTB herd breakdowns. These provide additional post-mortem data for SICCT test-negative cattle, allowing a rare opportunity to calculate the animal-level sensitivity of the test *relative* to the total number of SICCT test-positive and negative VL animals identified post-mortem (rSe). In this study, data were analysed from 16 whole herd slaughters (748 SICCT test-positive and 1031 SICCT test-negative cattle) conducted in GB between 1988 and 2010, using a Bayesian hierarchical model. The overall rSe estimate of the SICCT test at the *severe* interpretation was 85% (95% credible interval [CI]: 78–91%), and at *standard* interpretation was 81% (95% CI: 70–89%). These estimates are more robust than those previously reported in GB due to inclusion of post-mortem data from SICCT test-negative cattle.

## Introduction

Bovine tuberculosis (bTB), caused by *Mycobacterium bovis*, is a zoonotic disease that is endemic in many countries worldwide, impacting on animal health, welfare, productivity and trade. In Great Britain (GB), despite a compulsory test-and-slaughter programme introduced in 1950, the disease is now endemic in West and South-west England and also in Wales (although Scotland was declared officially bTB free in 2009). Bovine TB has been described as GB’s “*biggest endemic animal health issue*” [1] and in 2010–2011, the Department for the Environment, Food and Rural Affairs (Defra) spent over £91 million on bTB control in England [2]. The imperfect sensitivity (the probability that an infected animal is detected by the test) of the current diagnostic field test is one plausible contributor to the sustained increase in the incidence of bTB in GB over the last 25 years.

The intradermal tuberculin test is recognised by the World Organisation for Animal Health (OIE) and the European Commission as the primary screening test for detection of bTB in cattle [3], [4]. The application of this test, supplemented with slaughterhouse surveillance and movement controls on infected and suspect herds, underpins many bTB control programmes worldwide and, in the absence of wildlife reservoirs for *M. bovis*, has led to the successful eradication of bTB in many countries [4]–[6].

Two variants of the intradermal tuberculin test are in use today: the single intradermal tuberculin test, which measures a cell-mediated delayed-type hypersensitivity response to injection of *M. bovis* tuberculin in the caudal skin fold (e.g. New Zealand, USA) or the skin of the mid-cervical region (e.g. most European Union Member States); and the single intradermal comparative cervical tuberculin (SICCT) test, which compares immune responses to *M. bovis* (bovine) and *M. avium* (avian) tuberculins in the cervical region. In GB, Ireland and Portugal, the more specific, but less sensitive, SICCT test is used to minimise the likelihood of false positive results caused by exposure to cross-reacting mycobacteria [7], [8].

Nearly half of all cattle herds in GB are tested for bTB every year, 41% are tested every four years and the remainder tested at 2- or 3-yearly intervals (Defra, unpublished data, 2012). Cattle with a positive SICCT test result (known as “reactors”), or those from which *M. bovis* is cultured from suspect bTB lesions detected during commercial slaughter, trigger a bTB herd “breakdown”. All reactors are compulsorily slaughtered, whilst the affected herd is placed under movement restrictions and then re-tested at a minimum interval of 60 days (short-interval testing) until a series of tests with negative results is obtained. The number of short-interval tests, and their interpretation, is largely (but not exclusively) dictated by the detection of evidence of *M. bovis* infection at post-mortem examination (VLs) or on culture.

The sensitivity of the SICCT test is a measure of its ability to detect infected animals. Knowledge of SICCT test sensitivity is fundamental to understanding the potential success and limitations of bTB control programmes. Reduced test sensitivity increases the risk of within-herd persistence of infection, recurrent breakdowns, onward transmission through cattle movements and, potentially, infection spill-over into local wildlife reservoirs. Given the chronic nature of bTB and the relative insensitivity of post-mortem techniques used to confirm the presence of *M. bovis*, the true infection status of every animal cannot be determined and thus sensitivities have been estimated *relative* to post-mortem confirmation methods, namely detection of typical lesions of bTB (VLs) and/or laboratory culture of *M.* *bovis*.

SICCT test sensitivities in different countries and settings have been reported to range from 75.0–95.5% [8] and 77–95% [9], but for the majority of the studies from which these estimates were reported or derived, post-mortem data for a representative group of SICCT test-negative animals from the same population were lacking. Indeed, post-mortem data are rarely available for SICCT test-negatives, as normally only reactor animals are compulsorily slaughtered during herd breakdowns. Conducting experimental studies to collect such data would be expensive, labour-intensive and suffer from problems of extrapolation to the field situation. Without these data, the true proportion of infected animals not detectable by the test (false negatives) remains unknown, which could substantially impact on estimated values.

Whole herd slaughter (“stamping out” or depopulation) is a last-resort control option in GB, reserved only for severely or chronically infected herds in which repeated SICCT testing (occasionally supplemented since 2003 by interferon-gamma parallel testing [10]) has failed to resolve the breakdown. Depopulated herds provide a rare opportunity to improve estimates of relative SICCT test sensitivity (rSe), since they generate data on both SICCT test-positive *and* SICCT test-negative animals. In this study we analyse data from 16 such herds in GB, comprising a total of 748 SICCT test-positive and 1031 SICCT test-negative animals slaughtered between 1988 and 2010.

## Results

### Descriptive Results

Sixteen British cattle herds that underwent depopulation because of bTB infection between 1988 and 2010, and met the five inclusion criteria, were identified from VetNet (Table S1). A total of 748 reactors and 1031 direct contacts were slaughtered in those herds: the median number of reactors (per herd) was 34 (interquartile range [IQR] 23–45) and the median number of direct contacts removed was 53 (IQR 38–58.5). There were eleven beef and five dairy herds and the median number of animals slaughtered during the breakdown was 93 (IQR 58–128). The median herd size (of the maximum herd size recorded during the breakdown) was 82 (IQR 57–113).

Seven out of the 16 selected herds had at least one inconclusive reactor (IR: borderline test result) recorded during the breakdown. For five of these herds, all IRs were slaughtered (either immediately or after being retested later in the breakdown). Two herds had IRs recorded which were not all slaughtered but represented only a small percentage of the maximum herd size during the breakdown (1.3 and 5.4% respectively). Three breakdowns were triggered by *M. bovis* culture-positive lesions detected during routine slaughterhouse surveillance. These animals (infected but not SICCT-tested prior to slaughter), which represented between 1 and 8% of the number of animals slaughtered in each herd respectively, were excluded from the rSe calculations, as described in the Materials and Methods section.

### rSe Calculations

**Figure 1:**
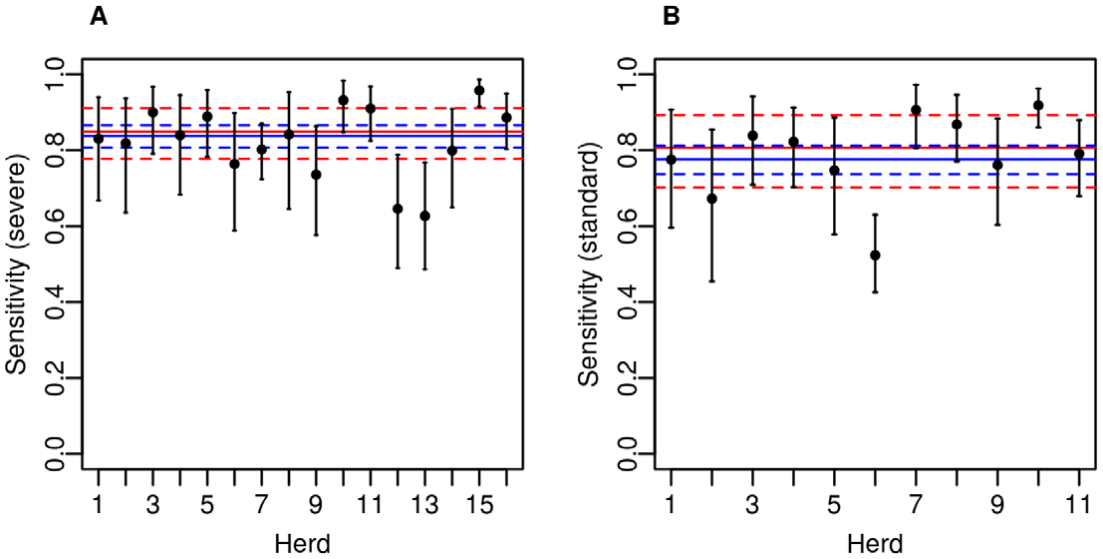
Overall mean *relative* Single Intradermal Comparative Cervical Tuberculin test sensitivity (rSe) estimates for each herd. Estimates are shown for severe and standard test interpretation, derived from logistic regression models fitted with and without herd-level effects. The solid lines represent the *mean* estimates, and the dashed lines represent the 95% credible intervals. The red lines are from the model *including* herd-level effects, and the blue lines are from the model *excluding* herd-level effects.

The overall rSe of the SICCT test at severe interpretation for the 16 slaughtered herds was estimated to be 85% (95% CI: 78–91%) from the model fitted with herd-level effects, and 84% (95% CI: 81–87%) when calculated assuming no herd-level heterogeneity (Table 1 and Figure 1A). Although data were available on skin test positivity or negativity for all 16 herds, data on exact skin test reaction sizes were only available for 11 of these (the five herds for which these data were not available had a breakdown date prior to September 2000, before which these data were not routinely recorded). When recalculated at the *standard* interpretation, the overall rSe was estimated to be 81% (95% CI: 70–89%) from the model fitted with herd-level effects, and 78% (95% CI: 74–81%) when calculated without (Table 1 and Figure 1B). This estimate is based on 11 herds only. The numbers of reactors at both interpretations are provided in Tables S2 and S3.

**Table 1:** Overall mean *relative* Single Intradermal Comparative Cervical Tuberculin (SICCT) test sensitivity (rSe) estimates (%).

	SICCT test interpretation#	Model *with* herd-level effects	Model *without* herd-level effects
**Overall mean ** ***relative*** ** sensitivity (95% CI)**	Severe	85 (78–91)	84 (81–87)
	Standard	81 (70–89)	78 (74–81)

# At severe test interpretation, results are based on 16 herds. At standard interpretation, results are based on 11 herds. 95% CI represent the 95% credible intervals.

Estimates are relative to visible lesion (VL) detection.

Figures 1A and 1B show the between-herd heterogeneity in the overall rSe estimates at severe and standard interpretation, obtained from the hierarchical model. Overall adjusted rSe estimates were slightly higher when the herd-level effects were included at both severe and standard interpretation, as a result of accounting for the herd-level heterogeneity. Although exceeding 90% in some herds, the estimated relative sensitivity of the test at severe interpretation was as low as 60% in others (Figure 1A). At the standard interpretation, one herd had a sensitivity estimate as low as 52% (Figure 1B).

Since we do not have SICCT test results for those animals that were detected via slaughterhouse surveillance, we conducted a sensitivity analysis assuming in the first instance that all of these animals would have been SICCT test positive, and in the second case assuming they would have been SICCT test negative. The results from these two extreme scenarios are shown in Table S4 and show negligible impacts on the estimates.

### Sensitivity of VL Detection Relative to Culture

Two of the slaughtered herds had culture data available for >90% of the animals slaughtered, in addition to post-mortem VL/NVL data. The rSe estimates for VL detection relative to culture for the two herds were 79% and 95% respectively, with an overall estimate of 90% (95% CI: 82–96%) [fitted without herd-level effects due to a lack of convergence when herd-level effects were included].

## Discussion

The SICCT test remains the primary bTB surveillance tool in several countries worldwide. Elsewhere, it is used as a second-line confirmatory (serial) test of caudal-fold and mid-cervical test reactors. The application of this test is central to restoring the officially TB-free status of infected herds in GB. A dearth of post-mortem data for SICCT test-negative animals has hindered the generation of reliable estimates for SICCT rSe. Depopulation, a radical bTB control method for severely affected herds, generates data for positive *and* negative-testing animals, providing a rare opportunity to improve current rSe estimates. Data for 16 cattle holdings, depopulated in GB between 1988 and 2010 due to bTB, were analysed calculating rSe relative to post-mortem detection of typical lesions of bTB (VLs) in slaughtered animals. The overall rSe was estimated to be 85% (95% CI: 78–91%) at the severe interpretation (16 herds) and 81% (95% CI: 70–89%) at the standard interpretation (11 herds) after accounting for herd-level heterogeneity.

Previously-reported median sensitivity estimates of the SICCT test at the standard (77.5%) and severe (92.2%) interpretation, were derived from a range of published studies that varied in location (some outside GB), tuberculin source and dosage [8] (these values were reported as 80% and 94% respectively; however, when the Costello *et al.* estimate [11] was re-examined, it was noted that this should have been included in the estimate for the *severe* interpretation, and thus the values have been recalculated to take account of this). For the five studies [11]–[15] where the test was performed as in GB, estimates ranged from 75.0–95.5% at the standard interpretation [8]. When the estimate from Costello *et al.* (1997) is excluded (since it was not interpreted at standard interpretation–as discussed previously), the range remains the same. SICCT test sensitivity ranged from 77–95% in an earlier review [9]: three of these studies [13]–[15] were included in the review by de la Rua-Domenech et al. (2006), and the remaining four studies [16]–[19] were not comparable, employing tuberculin extracted from *M. tuberculosis* that was replaced in GB by the current (*M. bovis*-derived) tuberculin in 1975 [9], [13].

Critically, the majority of these previous studies have lacked post-mortem data for SICCT-negative animals, which could plausibly have inflated the estimated sensitivities by reducing the denominator. However, one study carried out in Ireland [11], did slaughter the entire SICCT-tested study population (2528 animals), including all test-negatives, and also calculated sensitivity relative to VL detection (rSe). The rSe reported was 90.9% (severe interpretation), which is higher than our overall estimate although just inside our upper credible interval limit (overall mean 85%, 95% CI: 78–91%). The avian and bovine tuberculins used in the Costello study, manufactured in Lelystad (Netherlands), have been shown to be associated with a slightly higher probability of confirmation (VLs and/or culture) when compared with tuberculin manufactured in Weybridge (UK) [20], the SICCT test antigen used in the majority (69%) of the herds in our study (for the remaining herds, 25% herds used Lelystad and 6% herds [1 herd] had the tuberculin type classified as undetermined). Costello *et al.* also calculated sensitivity relative to VL detection, and although their higher rSe could have been due to a reportedly more thorough post-mortem inspection, this was not reflected in the percentage of reactors that had VLs: 41% of reactors and IRs in their study compared with 40% of reactors nationally in GB [21], although inclusion of IRs here could have artificially lowered this figure due to the proportion of VL animals being lower in IRs than in reactors (in both GB and Ireland).

The true infection status of all individual animals is impossible to determine. Sensitivities calculated in this study from naturally-infected herds were *relative* to detection of gross bTB lesions post-mortem. While in some respects our estimates may provide a lower bound for the true sensitivity, the impact of well-developed disease on lesion visibility in some of these herds is hard to determine as there may be biases in the depopulation data when compared to other situations, which could influence the rSe estimate in an unpredictable manner.

Any culture data that were available from the selected herds were not combined with the post-mortem VL data for rSe calculations, as there may have been bias towards reactors being more likely to be cultured than direct contacts. Sensitivity of VL detection, *relative* to culture positivity, was explored for two depopulated herds that had VL/NVL *and* culture data for >90% of animals slaughtered. The overall rSe derived from a logistic regression model was 90%, suggesting that VL detection is a good measure of infection status, as defined by detection by culture. This is supported by the fact that, in GB, an estimated 95% of all VL reactors yield *M. bovis* on culture [21].

Although in our calculations we do not assume that visible lesions are synonymous with infection, the majority of suspect lesions do indeed culture *M. bovis*. In 2011, around 75% of suspect granulomas detected during routine slaughterhouse surveillance (i.e. not in reactor animals identified during a breakdown) cultured *M. bovis* (Defra, unpublished, 2012). However, this proportion of culture-positive lesions is likely to be much higher in severe bTB breakdowns with previously confirmed infection and a high prevalence of within-herd infection. VLs have been shown to develop as early as 17 days in experimentally-infected calves infected with doses to mimic naturally-occurring infection [22], (albeit in an experimental situation where postmortem inspection for visible lesions was conducted at a higher level of detail with serial slicing of target organs and lymph nodes), which is earlier than the minimum time taken to develop a detectable SICCT test response, reported to be 3–6 weeks after infection [8].

Mechanisms underlying imperfect skin test sensitivity include factors relating to the tuberculin source and potency, the operator’s adherence to the prescribed test protocol [23]–[25], the stage of *M. bovis* infection in the tested animal (very early or too advanced to mount a detectable hypersensitivity response to tuberculin) [9], and factors that can mask or suppress the cell-mediated immune response to tuberculin, such as anti-inflammatory drugs and simultaneous infections with other pathogens. In addition, a period of transient and partial desensitisation has been shown to occur shortly after tuberculin injection [26] and, although EU protocols recommend a minimum interval of 60 days between sequential skin tests [27], recent evidence has shown that repeated SICCT testing at 60-day intervals can result in a progressive reduction in the comparative bovine-avian tuberculin response in naturally infected cattle [28].

Although the rSe at standard interpretation calculated in our study is in line with previously reported estimates, the mean rSe at severe interpretation calculated by Costello *et al.* (1997) only just fell within the upper credible interval limit of our estimate. Although the overall sensitivity generated from our study was lower at standard compared to severe interpretation (as one might expect), the 95% CIs overlapped considerably. This may have been due to the small numbers of animals reclassified during the change in interpretation (possibly limited by the number of herds in the study). Although this may suggest that in the field there may be little additional gain in applying the more severe interpretation, the small number of herds in this study should be considered when attempting to extrapolate the findings. The degree of between–herd heterogeneity for the rSe estimates also suggests that caution should be exercised in estimation from single herd breakdowns.

Veterinarians, farmers and government place rely on the SICCT test to detect bTB through surveillance testing and to clear infection from breakdown herds. It should be noted that our rSe estimates (and those from previous studies) are calculated at the *animal*-level, which provide a lower bound for the *herd*-level sensitivity (the ability to detect infected herds as part of a surveillance programme). The results suggest that even with the increased sensitivity at the more severe interpretation, undetected infected cattle may remain in some breakdown herds that are officially released from movement restrictions.

Almost a quarter of all breakdowns that are de-restricted after a programme of repeated skin testing at 60-day intervals with negative results recur in the same herd within 12 months, and 38% recur within 24 months [29]. Re-infection from badgers has been postulated to be a key mechanism behind persistence of infection in cattle herds in areas of endemic bTB incidence [30] and is anecdotally thought to be responsible for recurrent breakdowns in those areas. However, the failure of the SICCT test to detect every infected animal should also be recognised, as well as considering how the test is applied, both nationally as well at as the individual-herd level.

In many parts of GB the skin test has worked well as a bTB surveillance and eradication tool, with Scotland, for instance, gaining officially TB-free status in 2009 [31] in the apparent absence of a wildlife reservoir of *M. bovis*. As herds became attested and more counties became accredited TB free in the 1960s and 1970s, TB herd testing frequency in GB was gradually relaxed since the blanket enforcement of annual testing in the 1950s. It is only over the last 10–15 years that the herd testing coverage has been increasing year-on-year, with the whole of Wales and West and SW England now on yearly testing, reflecting the endemic bTB situation in those regions of GB [32]. The sensitivity of the overall bTB testing regime in GB has recently been boosted by the wider deployment of the interferon-gamma blood test in known infected herds and the introduction of compulsory pre-movement skin testing since 2006 [33], by the earlier removal of cattle with repeat inconclusive SICCT test results since 2010 [34] and by additional testing of high risk breakdown herds since 2011. Substantial efforts continue to be made to address gaps in the bTB eradication programme in GB and enhance the overall ability to detect and control infection (including training and auditing of operators conducting skin tests on farms).

### Conclusions

No ante-mortem diagnostic test for bTB is perfect and the SICCT test is no exception. Over 100 years ago, Bang (1892: cited in Monaghan, 1994) wrote “*the tuberculin test is no more perfect than most things in the world. Sometimes it fails, but it would be the greatest folly to reject this method because it is not able to give everything we desire*”. We have robustly estimated the overall rSe of the SICCT test, as applied in GB, and found it to be consistent with previous estimates at both standard and severe interpretations, but with some noticeable variation between individual herds. This information should form a key component when developing control and eradication plans and in evaluating their potential success.

## Materials and Methods

A national animal disease surveillance database (VetNet) recorded information relating to bTB testing and herd breakdowns in GB [35]. (This has now been replaced by a new IT system [Sam] since September 2011.) For each herd, this includes the ante-mortem SICCT test results for animals classed as reactors (R: positive), or inconclusive (IR: borderline result) during a bTB breakdown, and the post-mortem results i.e. whether typical lesions were detected on post-mortem inspection (or during further laboratory examination) for all cattle slaughtered to control the infection. In addition, these data are also available for at-risk cattle compulsorily removed as SICCT test-negatives (direct contacts), as is the case in partial or whole-herd depopulations.

### Identification of Whole Herd Slaughters

VetNet data were analysed up until 29^th^ October 2010. As bTB-infected holdings undergoing depopulation are not explicitly identifiable in this database, bTB breakdowns were selected for the study if they met *all five* of the following criteria:

1. **The presence of** ***M. bovis*** **infection had been confirmed** in the herd by detection of VLs and/or culture of the bacterium from at least one animal in the herd slaughtered during the breakdown.
2. **The percentage of the herd slaughtered was >90%.** The denominator used here is the *maximum* herd size recorded during the breakdown and all animals slaughtered over the entire duration of the breakdown were included in the numerator. Herd size may fluctuate between tests due to births/deaths or cattle moved (under licence) onto the farm/removed for bTB control, making it possible for the number of animals slaughtered to exceed the maximum herd size. Thus, those breakdowns in which the calculated percentage was >100% were not immediately excluded from the study.
3. **>90%** **of the herd size recorded at the last test during the breakdown was removed to slaughter.**
4. **The percentage of the animals slaughtered in the course of the breakdown that had a post-mortem (VL/NVL) result was >90%.** This cut-off was chosen to minimise the effect of missing VL/NVL data. This information should be recorded for all animals slaughtered, but it may be missing from the database on rare occasions.
5. **The number of animals slaughtered during the breakdown was >50.** This criterion was set to minimize spurious effects from small within-herd sample sizes.

Where the ancillary parallel interferon-gamma blood test had been performed in a herd (to accelerate the detection of infection in cattle that had tested negative to the SICCT test), any additional animals slaughtered as interferon-gamma positives were deemed to be SICCT test negative.

### Relative Test Sensitivity (rSe) Calculations

Culture of tissue samples is only conducted in GB in a representative sample of all the cattle slaughtered in a bTB breakdown or herd depopulation, thus preventing the inclusion of culture-positive cattle in the sensitivity calculations. In this study, SICCT test sensitivity for each herd was estimated *relative* (rSe) to the number of cattle presenting with typical tuberculous lesions during post-mortem carried out by veterinary and meat inspectors in approved slaughterhouses (the reference standard).

The SICCT test was conducted and its results interpreted according to the protocol set out at Annex B of EU Directive 64/432/EEC [27]. Slaughtered animals were recorded in VetNet as reactors, IRs or direct contacts. Any IRs slaughtered in the course of a breakdown as reactors/direct contacts/repeat IRs, were included in the rSe calculations if they had VLs (IRs with VLs were classified as SICCT test-positive). Untested cattle from eligible herds that had triggered a breakdown following detection of culture-positive bTB lesions during routine slaughterhouse surveillance were excluded, as no information on the SICCT test result was available for such animals.

In GB, SICCT test results can be interpreted using two different cut-offs for reactors, known as *standard* or *severe* interpretation [7]. For normal surveillance testing and in any bTB breakdowns without post-mortem evidence of *M. bovis* infection, so-called *unconfirmed* breakdowns, a standard interpretation is used whereby cattle with a positive *M. bovis* tuberculin reaction >4 mm greater than a positive or negative avian tuberculin reaction are classified as reactors, and those with a *M. bovis* tuberculin reaction exceeding the avian reaction by 1 to 4 mm are deemed IRs. In breakdowns with post-mortem or laboratory evidence of infection, so-called *confirmed* breakdowns, a severe interpretation is used whereby cattle are removed if they have a positive bovine reaction that exceeds the avian reaction by more than 2 mm (or where a positive bovine and a negative avian reaction are measured). Consequently, the standard interpretation IRs with the strongest bovine-avian tuberculin reaction differences (i.e. more than 2 mm and up to 4 mm) are culled as reactors under the severe interpretation of the SICCT test. Severe interpretation is therefore applied in an effort to accelerate the detection of infected cattle by increasing the sensitivity at the expense of the specificity.

On infected holdings that undergo depopulation due to bTB, the vast majority of SICCT tests will have been interpreted at severe interpretation. The overall rSe for this interpretation was calculated using a logistic regression model incorporating an individual herd-level effect to account for between-herd heterogeneity, such that:
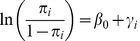
(1)Where 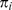 is the true unknown relative sensitivity for herd *i* where 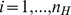. We fit this model in a Bayesian framework, and in order to complete the specification we give the intercept parameter 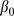 a vague N(0,100) prior distribution, and the herd-level effects, ***γ***, N(0,1/*τ*) prior distributions with the precision 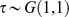. We note that this hierarchical model is analogous to a random intercepts model in a frequentist framework (though we avoid the use of this terminology since in the Bayesian framework all parameters are considered random). For comparison, we fit the model with and without the herd-level effects.

For each herd, the observed relative sensitivity for herd *i*, is 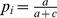, where *a* is the number of animals that were SICCT test-positive *and* had VLs post-mortem, and *a+c* is the total number of animals that had VLs post-mortem. The overall mean rSe is then 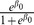. Fitting the model with the herd-level effects allows assessment of the degree of between-herd heterogeneity. For comparison, the sensitivity can then be recalculated without the herd-level effects. In addition, the rSe can be calculated using a *standard* SICCT test interpretation. IR cattle that were deemed reactors under the severe interpretation of the test were not classified as reactors under standard interpretation.

The models were fitted using Markov chain Monte Carlo (MCMC), and in each case a burn-in of 2500 iterations was used, followed by 10000 updates with the posterior distributions thinned to return 1000 samples. The overall (mean) estimate and 95% credible interval (CI) are reported to 2 significant figures (s.f.).

### Culture Data

Although culture is not routinely conducted for every animal in a breakdown, on those occasions where culture *and* VL/NVL data were available for >90% animals slaughtered in a herd, the rSe of VL detection was measured against a reference standard of culture positivity using equation (1) as before. This was possible in only two herds.

Analyses were carried out using the R statistical language [36], except the fitting of the Bayesian model which was conducted in WinBUGS [37] using the R2WinBUGS package [38].

## Supporting Information

Table S1 **Descriptive data of herds identified as whole herd slaughters.**
(DOCX)

Table S2 **Data for calculating Single Intradermal Comparative Cervical Tuberculin (SICCT) test sensitivity at the** ***severe*** **interpretation.**
(DOCX)

Table S3 **Data for calculating Single Intradermal Comparative Cervical Tuberculin (SICCT) test sensitivity at the** ***standard*** **interpretation.**
(DOCX)

Table S4 **Sensitivity analysis assuming extreme cases for slaughterhouse animals that were not tested. Values are posterior means (95% credible intervals).**
(DOCX)
