# Supplementary material for: Estimation of the Relative Sensitivity of the Comparative Tuberculin Skin Test in Tuberculous Cattle Herds Subjected to Depopulation
Source: PLoS One. 2012 Aug 21;7(8):e43217. doi: 10.1371/journal.pone.0043217 (PMC3424237; doi:10.1371/journal.pone.0043217)
Supplement: Table S1 — Descriptive data of herds identified as whole herd slaughters. (DOCX) [file pone.0043217.s001.docx]

**Table S1. Descriptive data of herds identified as whole herd slaughters.**

| Herd ID | Year breakdown started | Breakdown duration (days) | Herd type | Herd size | No.  slaughtered during breakdown | % with VL/NVL data | No. reactors (with skin measures) | No. direct contacts  (with skin measures) | No. IR ^§^ | No. slaughterhouse cases | % slaughtered that were direct contacts |
| --- | --- | --- | --- | --- | --- | --- | --- | --- | --- | --- | --- |
| 1 | 2007 | 661 | Dairy | 56 | 59 | 95 | 33 (33) | 26 (26) | 0 | 0 | 44 |
| 2 | 2006 | 265 | Dairy | 86 | 96 | 96 | 39 (38) | 57 (54) | 0 | 0 | 59 |
| 3 | 2008 | 365 | Beef | 49 | 52 | 100 | 34 (28) | 16 (16) | 0 | 2 | 31 |
| 4^¶^ | 1988 | 120 | Beef | 56 | 56 | 100 | 23 (0) | 33 (0) | 0 | 0 | 59 |
| 5^¶^ | 2002 | 219 | Dairy | 109 | 127 | 98 | 41 (41) | 85 (85) | 1 | 0 | 67 |
| 6 | 2005 | 99 | Beef | 57 | 56 | 100 | 18 (18) | 38 (38) | 0 | 0 | 68 |
| 7 | 2003 | 709 | Dairy | 220 | 379 | 91 | 167(167) | 212 (186) | 0 | 0 | 56 |
| 8 | 1998 | 188 | Beef | 49 | 55 | 100 | 16 (0) | 39 (0) | 0 | 0 | 71 |
| 9 | 1996 | 114 | Beef | 74 | 80 | 100 | 22 (0) | 52 (0) | 0 | 6 | 65 |
| 10 | 2003 | 348 | Beef | 92 | 97 | 92 | 42 (42) | 54 (54) | 0 | 1 | 56 |
| 11 | 2004 | 259 | Dairy | 78 | 93 | 100 | 55 (55) | 38 (38) | 0 | 0 | 41 |
| 12 | 1997 | 246 | Beef | 177 | 180 | 99 | 24 (0) | 156 (0) | 0 | 0 | 87 |
| 13 | 1995 | 242 | Beef | 91 | 92 | 99 | 23 (0) | 63 (0) | 6 | 0 | 68 |
| 14 | 2004 | 254 | Beef | 74 | 77 | 100 | 23 (23) | 54 (54) | 0 | 0 | 70 |
| 15 | 2010 | Ongoing >240 | Beef | 164 | 157 | 93 | 110 (110) | 57 (57) | 0 | 0 | 36 |
| 16 | 2010 | Ongoing >240 | Beef | 125 | 129 | 99 | 78 (78) | 51 (51) | 0 | 0 | 40 |
| **TOTAL** |  |  |  |  |  |  | **748** | **1031** | **7** | **9** |  |

VL = visible lesions. NVL = no visible lesions. IR = inconclusive reactor. ^§^ IRs identified during the breakdown and retested were mostly recorded as reactors or direct contacts in VetNet when slaughtered. However, there were some occasions where the animal, although slaughtered, was still recorded as an IR. The number of these is shown in this column. These were excluded from calculations as they had not been SICCT tested. ^¶^ Herds for which culture data were available.
