# Supplementary material for: Estimation of the Relative Sensitivity of the Comparative Tuberculin Skin Test in Tuberculous Cattle Herds Subjected to Depopulation
Source: PLoS One. 2012 Aug 21;7(8):e43217. doi: 10.1371/journal.pone.0043217 (PMC3424237; doi:10.1371/journal.pone.0043217)
Supplement: Table S2 — Data for calculating Single Intradermal Comparative Cervical Tuberculin (SICCT) test sensitivity at the severe interpretation. (DOCX) [file pone.0043217.s002.docx]

**Table S2. Data for calculating Single Intradermal Comparative Cervical Tuberculin (SICCT) test sensitivity at the *severe* interpretation.**

| **Herd ID** | **No. reactors with VL** | **No. direct contacts with VL** | **Total no. VL** | **No. direct contacts with NVL** | **No. reactors with NVL** | **Total NVL** |
| --- | --- | --- | --- | --- | --- | --- |
| 1 | 14 | 3 | 17 | 23 | 16 | 39 |
| 2 | 12 | 3 | 15 | 52 | 25 | 77 |
| 3 | 25 | 2 | 27 | 14 | 9 | 23 |
| 4^#^ | 16 | 3 | 19 | 30 | 7 | 37 |
| 5 | 37 | 4 | 41 | 78 | 5 | 83 |
| 6 | 13 | 5 | 18 | 33 | 5 | 38 |
| 7 | 75 | 19 | 94 | 175 | 76 | 251 |
| 8^#^ | 11 | 2 | 13 | 37 | 5 | 42 |
| 9^#^ | 21 | 9 | 30 | 43 | 1 | 44 |
| 10 | 35 | 1 | 36 | 44 | 5 | 49 |
| 11 | 41 | 3 | 44 | 35 | 14 | 49 |
| 12^#^ | 21 | 14 | 35 | 141 | 3 | 144 |
| 13^#^ | 24 | 17 | 41 | 45 | 5 | 50 |
| 14 | 22 | 6 | 28 | 48 | 1 | 49 |
| 15 | 92 | 2 | 94 | 34 | 18 | 52 |
| 16 | 51 | 6 | 57 | 44 | 27 | 71 |
| **TOTAL** | **510** | **99** | **609** | **876** | **222** | **1098** |

VL = visible lesions. NVL = no visible lesions. ^#^ Breakdowns excluded from calculations at *standard* interpretation.
