# Supplementary material for: Estimation of the Relative Sensitivity of the Comparative Tuberculin Skin Test in Tuberculous Cattle Herds Subjected to Depopulation
Source: PLoS One. 2012 Aug 21;7(8):e43217. doi: 10.1371/journal.pone.0043217 (PMC3424237; doi:10.1371/journal.pone.0043217)
Supplement: Table S3 — Data for calculating Single Intradermal Comparative Cervical Tuberculin (SICCT) test sensitivity at the standard interpretation. (DOCX) [file pone.0043217.s003.docx]

**Table S3. Data for calculating Single Intradermal Comparative Cervical Tuberculin (SICCT) test sensitivity at the *standard* interpretation.**

| **Herd ID** | **No. reactors with VL** | **No. direct contacts with VL** | **Total no. VL** | **%VLs with skin mm recorded** | **No. direct contacts with NVL** | **No. reactors with NVL** | **Total NVL** |
| --- | --- | --- | --- | --- | --- | --- | --- |
| 1 | 13 | 4 | 17 | 100 | 23 | 16 | 39 |
| 2 | 9 | 6 | 15 | 93 | 68 | 9 | 77 |
| 3 | 23 | 4 | 27 | 93 | 20 | 3 | 23 |
| 5 | 34 | 7 | 41 | 100 | 79 | 4 | 83 |
| 6 | 13 | 5 | 18 | 100 | 33 | 5 | 38 |
| 7 | 47 | 47 | 94 | 93 | 210 | 41 | 251 |
| 10 | 34 | 2 | 36 | 100 | 45 | 4 | 49 |
| 11 | 39 | 5 | 44 | 100 | 36 | 13 | 49 |
| 14 | 21 | 7 | 28 | 100 | 48 | 1 | 49 |
| 15 | 88 | 6 | 94 | 100 | 34 | 18 | 52 |
| 16 | 45 | 12 | 57 | 100 | 48 | 23 | 71 |
| **TOTAL** | **366** | **105** | **471** |  | **644** | **137** | **781** |

VL = visible lesions. NVL = no visible lesions.
