# Supplementary material for: Estimation of the Relative Sensitivity of the Comparative Tuberculin Skin Test in Tuberculous Cattle Herds Subjected to Depopulation
Source: PLoS One. 2012 Aug 21;7(8):e43217. doi: 10.1371/journal.pone.0043217 (PMC3424237; doi:10.1371/journal.pone.0043217)
Supplement: Table S4 — Sensitivity analysis assuming extreme cases for slaughterhouse animals that were not tested. Values are posterior means (95% credible intervals). (DOCX) [file pone.0043217.s004.docx]

**Table S4. Sensitivity analysis assuming extreme cases for slaughterhouse animals that were not tested. Values are posterior means (95% credible intervals).**

| **Severe interpretation** | **Model *with* herd-level effects** | **Model *without* herd-level effects** |
| --- | --- | --- |
| Model excluding SLH cases (as reported in paper) | 85 (78-91) | 84 (81-87) |
| Model including SLH cases (assumed to be SICCT test positive) | 85 (79-91) | 84 (81-87) |
| Model including SLH cases (assumed to be SICCT test negative) | 84 (76-90) | 83 (80-85) |
